# A case of leiomyosarcoma of the ovarian vein with obstructive uropathy and hepatic metastasis

**DOI:** 10.4102/sajr.v26i1.2501

**Published:** 2022-10-28

**Authors:** Sanjay M. Khaladkar, Tejvir Singh, Karthik Mohanan, Rajesh Kuber, Satvik Dhirawani

**Affiliations:** 1 Department of Radiodiagnosis, Dr. D. Y. Patil Medical College, Hospital and Research Centre, Pune, India

**Keywords:** leiomyosarcoma, gonadal vein, ovarian vein, retroperitoneal mass, vascular origin, claw sign, obstructive uropathy, hepatic metastasis

## Abstract

Leiomyosarcoma (LMS) is a smooth muscle-derived malignant mesenchymal tumour. Primary LMS arising from the ovarian vein is uncommon. A 51-year-old female presented with left loin pain and was diagnosed with a LMS arising from the gonadal vein. The patient refused any treatment. Early detection and timely diagnosis of retroperitoneal masses will improve the prognosis and survival rate in these patients. CT imaging plays an important role in diagnosis.

**Contribution:**

Retroperitoneal leiomyosarcoma of vascular origin is a rare entity. CT plays crucial role in diagnosing them by demonstrating the extent of the tumor along the gonadal vein. Early detection and timely diagnosis of retroperitoneal masses will improve the prognosis and survival rate in these patients.

## Introduction

Leiomyosarcoma (LMS) is a sarcoma derived from smooth muscles. It is a malignant mesenchymal tumour that may have a vascular or non-vascular origin. Primary LMS of vascular origin is uncommon, representing less than 1 in 100 000 of all malignant tumours.^1^

Venous LMSs account for 5% – 7% of soft tissue sarcomas. About 50% of cases arise from the inferior vena cava (IVC) and at least 200 cases have been reported to date.^2^ They can also arise from the renal, mesenteric, hepatic, saphenous or gonadal veins.^3^ The ovarian veins are an uncommon site of LMS - there have been 15 cases described in the literature to date.^4^ Leiomyosarcoma from other origins have a relatively better prognosis than LMS of vascular origin.^1^

This report describes a biopsy confirmed case of LMS arising from the left ovarian vein and discusses the imaging findings on ultrasonography and multidetector CT.

## Case report

**FIGURE 1:**
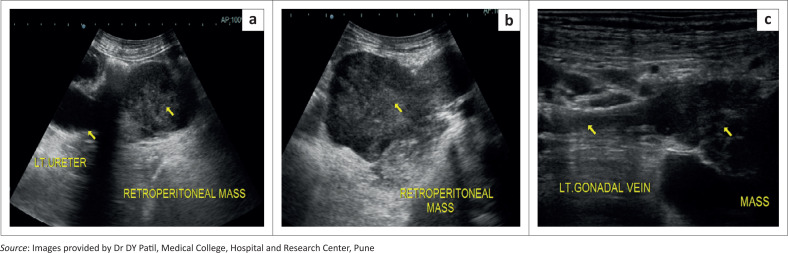
(a–c) Ultrasound abdomen showing retroperitoneal mass of heterogeneous echotexture along the course of the left gonadal vein involving adjoining left ureter with proximal hydroureter.

A 51-year-old female presented with left sided loin pain for 6 months. There was no history of urinary complaints, haematuria, weight loss, loss of appetite or cough. Ultrasonography of the abdomen and pelvis demonstrated a well-defined solid mass in the left retroperitoneum with a lobulated outline, anterolateral to the adjoining left psoas muscle, causing compression of the left upper ureter with resultant moderate proximal hydronephrosis and hydroureter (Figures 1a–c).

**FIGURE 2:**
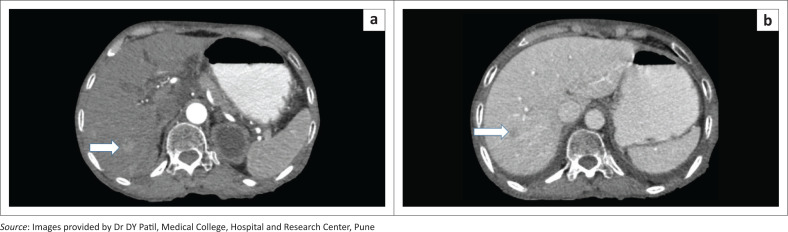
(a–b) Axial CT abdomen- arterial (a) and portal phase (b) revealing a hepatic metastasis (white arrow) in segment VI appearing hyperdense in the arterial and hypodense in the portal phase.

CT of the abdomen and pelvis revealed multiple well-defined lesions in the right hepatic lobe, with enhancement in the arterial phase, appearing hypodense in the portal phase and becoming isodense on venous and delayed phases – suggestive of hypervascular metastases. The largest lesion measured 16 mm × 12 mm in segment VI (Figures 2a, b).

**FIGURE 3:**
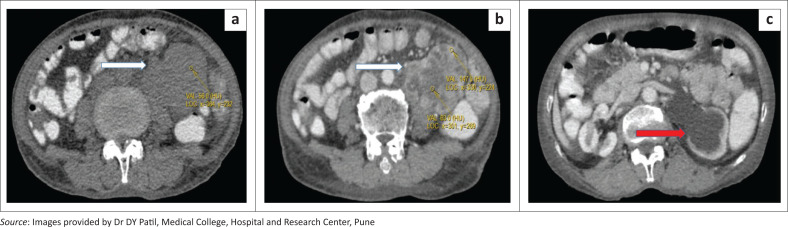
(a–c) Axial CT of abdomen showing a well defined solid retroperitoneal mass (white arrow) anterior to the left psoas and medial to the descending colon appearing isodense to muscle on pre-contrast (a), showing heterogeneous post contrast enhancement (b), causing proximal left hydronephrosis and hydroureter (red arrow) (c).

A large solid mass with a lobulated outline, measuring approximately 82 mm × 52 mm × 64 mm (craniocaudal × anteroposterior × transverse), was identified in the retroperitoneum along the anterior surface of the left psoas muscle from the L3 to the L5 levels, demonstrating heterogeneous post-contrast enhancement with central non-enhancing hypodense areas of necrosis. No calcification was observed. The fat plane between the mass and the left psoas muscle was obscured. The adjacent left ureter was markedly compressed and involved by the mass with resultant proximal obstructive uropathy (Figures 3a–c).

**FIGURE 4:**
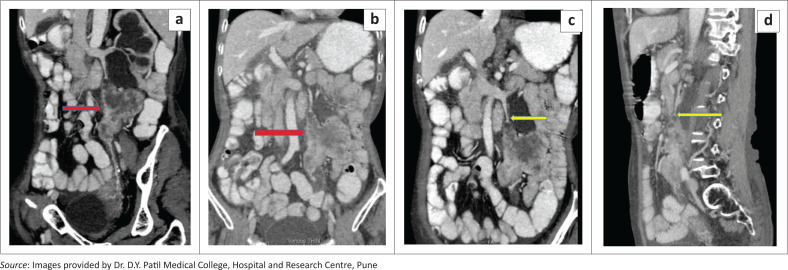
Coronal oblique (a), showing a well-defined heterogeneous mass (red arrow) along the course of left gonadal vein causing hydronephrosis and hydroureter. Coronal (b–c) and sagittal (d) reformatted CT abdomen reveal a well defined heterogeneously enhancing retroperitoneal mass (red arrow) along the course of the left gonadal vein causing filling defect (yellow arrow)(c–d).

The left gonadal vein revealed a filling defect at the L3 level. The mass appeared elongated and oblong-shaped along the course of the left gonadal vein, which was indistinctly visible from the L3 to the L5 levels. Mass effect was noted on the adjacent descending colon and small bowel loops with maintained intervening fat planes. The left kidney showed marked hydronephrosis with thinning of the renal parenchyma and a dilated renal pelvis with an AP diameter of 3 cm. The left ureter was dilated in its upper portion and could be traced up to the inferior endplate of the L3 vertebral body, where it appeared compressed and involved by the retroperitoneal mass. There was no excretion of contrast by the left kidney on the delayed phase (Figure 4a–d).

Mild ascites and small bilateral pleural effusions were noted. The rest of the abdomen was unremarkable. A diagnosis of a neoplastic retroperitoneal mass within the left gonadal vein was considered.

A needle aspirate sample taken from the retroperitoneal lesion revealed a tumour composed of interlacing bundles of spindle cells with mild atypia. Mitosis was observed at 3 to 4 per 10 high power field. A histological diagnosis of LMS was ascertained. The patient refused biopsy of the hepatic lesions.

The patient was advised to have surgery with radiotherapy and chemotherapy as adjuvant therapy but refused any treatment and demised two months later.

## Discussion

Leiomyosarcoma is the second most common retroperitoneal sarcoma in adults. Smooth muscles present in the walls of retroperitoneal veins or embryonic remnants are the sites of origin.^1^ The ovarian vein is the eighth most usually afflicted vessel among vascular LMS. Until now, there are six known cases of LMS (40%) from the left ovarian vein and nine cases of known LMS (60%) from the right ovarian vein that have been published.^4^

Retroperitoneal LMS has three major growth patterns: completely extravascular or extra luminal (62%), completely intravascular or intraluminal (5%) and a combination of extra luminal and intraluminal patterns (mixed – 33% of cases). Extra luminal LMS is usually detected late, while those with intraluminal and mixed patterns usually show early symptoms depending on the affected vein.^1^

Retroperitoneal LMS is typically solid with cystic areas related to necrosis. The characteristic imaging features of LMS arising from the gonadal vein are the presence of a solid or necrotic extra- or intra-luminal retroperitoneal mass, not arising from a retroperitoneal organ and its continuity with the enhancing gonadal vein along its course. Rarely, there may be extensive vascular proliferation and dilatation within the mass. In such cases, the differential would include haemangioma, haemangiopericytoma and angiolipoma.^5^

Contrast enhanced CT is superior to ultrasound and is the diagnostic test of choice for gonadal vein LMS. It is extremely useful in the preoperative diagnosis, assessing the boundaries and extent of the LMS.^6^ A large lobulated, retroperitoneal mass with heterogeneous enhancement, extending in a longitudinal direction along the course of gonadal vein, which may have internal necrotic components, is suggestive of the diagnosis. CT depicts the exact origin of mass from the vessel and excludes masses arising from the other retroperitoneal structures. With multidetector CT, multi-planar reconstruction is extremely useful in demonstrating the relationship of the mass to the vessel, the tumour extent, the relationship with adjacent structures and the presence of necrotic/cystic components and hypertrophied vessels within the mass. Demonstration of the claw sign indicates a vascular origin of the mass.^6^ Imaging with Fluorodeoxyglucose positron emission tomography (FDG-PET) will show increased uptake within the mass. Haematological metastases occur more commonly than lymphatic metastases, and may occur in the liver, lung, peritoneum and brain.^7^

Histopathological examination shows bundles of spindle-shaped cells, perinuclear vacuoles, eosinophilic cytoplasm and mitotic figures. Immunohistochemistry is positive for smooth cell actin, vimentin, desmin and negative for S-100 protein or neuron specific enolase.^3^ Up to 60% of adjacent organs are microscopically invaded by the tumour at pathology.^8^ *En bloc* resection with histopathological free margins is the surgical gold standard with good prognosis.^6,9^ Retroperitoneal sarcomas have a more than 50% recurrence rate and a 5-year-survival of 52% – 60%, despite complete resection.^10^ Consequently, adjuvant chemotherapy and radiotherapy is recommended.

Diagnosis can be incidental in a third of cases and is often delayed, resulting in a worse prognosis. Cases seldom present with symptoms unless they have progressed to a large size as with other retroperitoneal tumours.^3^

None of the previously published ovarian vein LMS cases reported hepatic metastases. Although the hepatic lesion in the current case was not biopsied, metastases were highly probable and portended a worse prognosis for the patient.

## Conclusion

Leiomyosarcoma arising from the gonadal vein is a rare retroperitoneal tumour and diagnosis is often delayed because of non-specific symptoms. It should be suspected when tumour is seen along the course of the gonadal vein and extends in a longitudinal manner. Both ultrasound and CT are useful in demonstrating the relationship of the mass with the gonadal vein along with its extent. Multidetector CT is useful in preoperative evaluation of the mass with respect to location, extent, growth patterns and the relationship of the tumour and vessel.
